# Growth responses of eight wetland species to water level fluctuation with different ranges and frequencies

**DOI:** 10.1371/journal.pone.0220231

**Published:** 2019-07-25

**Authors:** Guan-Wen Wei, Yue Chen, Xin-Sheng Sun, Yu-Han Chen, Fang-Li Luo, Fei-Hai Yu

**Affiliations:** 1 School of Nature Conservation, Beijing Forestry University, Beijing, China; 2 Institute of Wetland Ecology & Clone Ecology, Zhejiang Provincial Key Laboratory of Plant Evolutionary Ecology and Conservation, Taizhou University, Taizhou, Zhejiang, China; Shandong University, CHINA

## Abstract

Fluctuation range and frequency are two important components of water level fluctuation, but their effects on wetland plants have not been evaluated separately. We subjected eight wetland species to a control treatment with static water level and fluctuation treatments with different ranges or frequencies to examine their effects on plant growth. *Acorus calamus*, *Butomus umbellatus* and *Iris wilsonii* showed high survival rates in all treatments with various fluctuation ranges and frequencies. Their survival rates were higher at the medium fluctuation frequency than at the low and high frequencies, suggesting beneficial effects of the medium frequency. In the experiment comparing the fluctuation ranges, *A*. *calamus* and *I*. *wilsonii* could maintain the capacity for asexual propagation and accumulate higher biomass compared with the control plants, while biomass of the other six species dramatically decreased. In the experiment comparing fluctuation frequency, species with relatively high survival rates (≥ 50%) maintained or increased the capacity of asexual propagation, and *A*. *calamus* and *I*. *wilsonii* allocated relatively more biomass to roots, which may enhance plant growth and survival. In contrast, these species did not show increased biomass allocation to shoots in response to both fluctuation range and frequency, presumably because shoots are prone to mechanical damage caused by streaming floodwater. Taken together, biomass accumulation in roots rather than in shoots and the ability to asexually propagate are important for the survival of these species during water fluctuation.

## Introduction

Water level fluctuation is a major component of hydrological regimes, which is a common and crucial process encountered by wetland plants [1–2]. Frequent submergence and de-submergence during water fluctuation result in great variations in the availability of light, O_2_ and CO_2_ for plants [3–5]. Also, wave disturbance during water level fluctuation can impose mechanical damage to wetland plants [6–9]. Furthermore, water level fluctuation can lead to sediment resuspension and strongly decrease concentrations of soil nutrients in wetlands [10–11]. As a result, water fluctuation has strong impacts on plant growth and distribution [12–13]. Former studies have shown that moderate levels of water fluctuation could promote seed germination and seedling establishment, while intensive fluctuation could greatly restrict plant growth and distribution [14].

Water level fluctuation has two important components: fluctuation range and frequency [15–16]. The range of fluctuation refers to the difference between the lowest and the highest water level during a fluctuation cycle, and the frequency of fluctuation is defined by the number of fluctuation cycles (e.g. a cycle going from the lowest to the highest water level and then to the lowest again) within a certain period. Global climate change is predicted to increase the frequency of extreme precipitation events in most temperate regions [17]. Consequently, hydrological interactions between water body and the surrounding regions may be altered significantly, leading to the occurrence of more frequent water fluctuation affecting greater areas of wetlands [9, 18–20]. To develop strategies for wetland management in the face of such climate scenario, it is necessary to assess the effects of different ranges and frequencies of water level fluctuation on growth of wetland plants.

Plants subjected to larger fluctuation range experience lower O_2_ and light intensity, but higher CO_2_ and hydrostatic pressure [21–22]. Till now, many studies have focused on response of wetland plants to constant water depth, but not to different fluctuation ranges [23–26]. A few studies on plant response to fluctuation range have shown that wetland species are sensitive to large fluctuation range but may be able to adapt, to some extent, to smaller and moderate fluctuation ranges to gain more biomass in certain cases [13, 15, 22]. Still, phenotypic plasticity in response to fluctuation range has been relatively less studied [22, 27].

Higher fluctuation frequency means faster changes between submergence and de-submergence conditions, shortening each period of aerobic conditions [8, 16]. Plant species, which are slow in responding to changing environments, could not resume photosynthesis and thus growth within a short period [6, 28]. By contrast, plant species, which quickly respond to water level changes, often develop fragile shoots by promoting stem elongation and aerenchyma formation [29–30]. These fragile tissues, however, can be easily damaged by streaming water during water fluctuation [6–9]. Thus, resource and biomass investment in shoot may be more risky than the same investment in roots during water fluctuation. Our recent study has shown that recurrent submergence and de-submergence events during water fluctuation both impose negative effects on biomass accumulation of a flood-tolerant species, with severer effects seen at higher frequency [31]. Therefore, the capacity to maintain or even increase biomass accumulation may be important for plants to survive high-frequency water fluctuations. However, it is still not clear whether plant tolerance to high-frequency fluctuation is related to the ability to maintain biomass accumulation. Another question is related to biomass allocation: whether plants allocate more biomass to shoots, roots or reproduction for their survival.

Here we examined the effects of fluctuation range and frequency on growth and biomass allocation of eight wetland species in controlled experiments. These plants were subjected to a control treatment with static water level and fluctuation treatments with three different levels of fluctuation ranges or frequencies. Theoretical and empirical studies have shown that the intermediate level of disturbance is beneficial to plant growth compared with the low and the high levels [14, 16, 22]. We therefore hypothesized (1) that plants perform better at the medium level than at the low and the high levels of fluctuation range and frequency, and (2) that species with higher survival rates allocate relatively more biomass to maintain root growth and reproduction than to shoot elongation.

## Material and methods

### Plant species

Eight wetland plant species, which were neither endangered nor protected, were selected for the experiments: *Acorus calamus* L., *Butomus umbellatus* L., *Iris wilsonii* C. H. Wright, *Lythrum salicaria* L., *Polygonum hydropiper* L., *Pontederia cordata* L., *Sagittaria trifolia* L. and *Typha minima* Funck ex Hoppe (S1 Table). They are widely distributed in wetlands of China and largely affected by water fluctuation [8, 32]. For each species, seedlings of uniform size and similar developmental stages (S1 Table) were bought from Hongyun Aquatic Flower Base in Tianjin, China. The plants were cultivated at the ecological field station of Miyun Reservoir Experimental Base or Cuihu Lake Experimental Base in Beijing, China.

### Experimental design

#### Response to different fluctuation ranges (Exp. 1)

**Fig 1:**
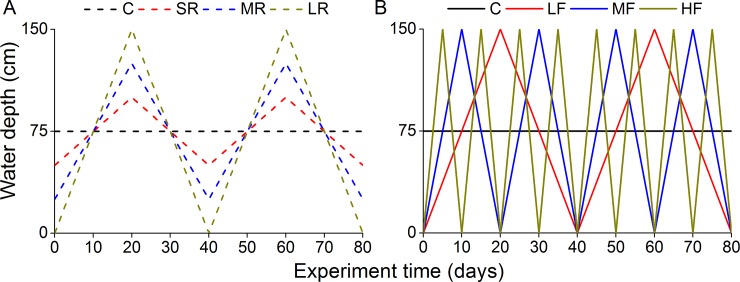
Settings of different water fluctuation ranges and frequencies. The treatments were: C–water depth was maintained constantly at 75 cm; SR–water depth changing between 50 and 100 cm twice (two cycles); MR–water depth changing between 25 and 125 cm twice; LR–water depth changing between 0 and 150 cm twice; LF–water depth changing between 0 and 150 cm twice; MF–water depth changing between 0 and 150 cm four times; HF–water depth changing between 0 and 150 cm eight times, during the 80-d experiment.

Seedlings were transferred to plastic pots (12.5 cm bottom diameter, 17 cm upper diameter and 18 cm deep) filled with local soil from Miyun Reservoir Experimental Base. After recovery for two weeks (8 June 2013), 24 plants from each species were randomly subjected to four treatments, with six replicates each. Thus, there were in total 192 plants for the eight species. The six replicates were separately placed in six tanks (155 cm diameter and 167 cm deep) as six blocks. According to our previous studies [16], four fluctuation ranges were: (i) control (C)–no fluctuation, constantly submerged in static water at 75 cm water depth (the vertical depth from soil surface to water surface), (ii) small range (SR)–water depth changing between 50 and 100 cm, (iii) medium range (MR)–between 25 and 125 cm, (iv) large range (LR)–between 0 and 150 cm (Fig 1A). As with the changes in fluctuation range, two successive fluctuation cycles were conducted during an 80-d experiment. The pots were suspended in the tanks by using ropes that were tied to a steel frame mounted on top of each tank. The water level fluctuation was applied by changing the vertical position of pots in the tank: releasing the rope increased the flooding depth whereas pulling up the rope decreased the flooding depth. The water depth was changed little by little every five days, mimicking gradual changes in water level.

#### Response to different fluctuation frequencies (Exp. 2)

On 19 June 2013, the Experiment II was started using a similar setup at Cuihu Lake Experimental Base, including eight species × four fluctuation frequencies × six replicates. According to our previous studies [8], four fluctuation frequencies were: (i) control (C)–no fluctuation, constantly submerged at 75 cm water depth, (ii) low frequency (LF)–water level fluctuating between 0 and 150 cm twice during the 80-d experiment (40 days per cycle), (iii) medium frequency (MF)–the same fluctuation range but four times (20 days per cycle), and (iv) high frequency (HF)–the same fluctuation range but eight times (10 days per cycle; Fig 1B). The flooding water used in both experiments was the tap water which was added during the experiment to compensate for water loss due to evaporation.

### Growth measurements

On day 80, all plants were taken out of the flooding tanks. Height and ramet number of each plant were measured. Height of a plant was defined as the length of the longest stem of the plant. After 10-d recovery following de-submergence (day 90), if leaves were still turgid and green and new leaves or buds grew out, the plant was considered alive [33]. The surviving plants were harvested on day 90, divided into roots and shoots and dried at 75°C for 72 h to determine dry weight.

### Data analyses

One-way ANOVA followed by Duncan test was used to test differences between treatments for each experiment. During the experiment, one replicate of the treatment of fluctuation range was damaged due to heavy rainfall; the related data were not used in the analyses. Before the analyses, all data were checked for homogeneity of variance. The effects were considered to be significant if *P* < 0.05. All analyses were performed by using SPSS 16.0 (SPSS, Chicago, IL, USA).

## Results

### Survival rate

At the end of the range experiment (Exp. 1), *A*. *calamus*, *B*. *umbellatus*, *I*. *wilsonii* and *S*. *trifolia* had relatively high survival rates (≥ 60%; Table 1A) while the survival rates of other four species were lower in most cases. Two species, *P*. *cordata* and *T*. *minima*, did not survive LR and/or MR treatment. At the end of the frequency experiment (Exp. 2), the four species, which had high survival rates in the Exp. 1 (*A*. *calamus*, *B*. *umbellatus*, *I*. *wilsonii* and *S*. *trifolia*), plus *L*. *salicaria* and *P*. *cordata* had relatively high survival rates (≥ 50%), although *L*. *salicaria* and *S*. *trifolia* seemed to suffer in HF (Table 1B). Compared to the control, the survival rate of *P*. *hydropiper* sharply decreased in the flooding treatments at all frequencies. Of all species examined, *A*. *calamus* was the best and *T*. *minima* the poorest survivor in both Exp. 1 and Exp. 2 (Table 1).

**Table 1:** The survival rate (%) of eight wetland species subjected to water level fluctuation with different ranges (A) and frequencies (B).

	(A) Fluctuation range				(B) Fluctuation frequency			
Species	C	SR	MR	LR	C	LF	MF	HF
*A*. *calamus*	100	100	100	100	83	83	100	83
*B*. *umbellatus*	100	80	100	100	100	50	100	50
*I*. *wilsonii*	80	100	100	100	50	67	100	50
*L*. *salicaria*	20	40	40	20	100	83	50	0
*P*. *cordata*	60	60	20	0	100	100	50	83
*P*. *hydropiper*	40	20	20	40	100	17	17	17
*S*. *trifolia*	60	60	60	60	67	83	83	0
*T*. *minima*	60	20	0	0	17	0	0	0

Treatment codes: C–control (static water level); SR, MR and LR for small, medium and large range of water level fluctuation, respectively; LF, MF and HF for low, medium and high frequency of water level fluctuation, respectively.

### Growth responses

**Fig 2:**
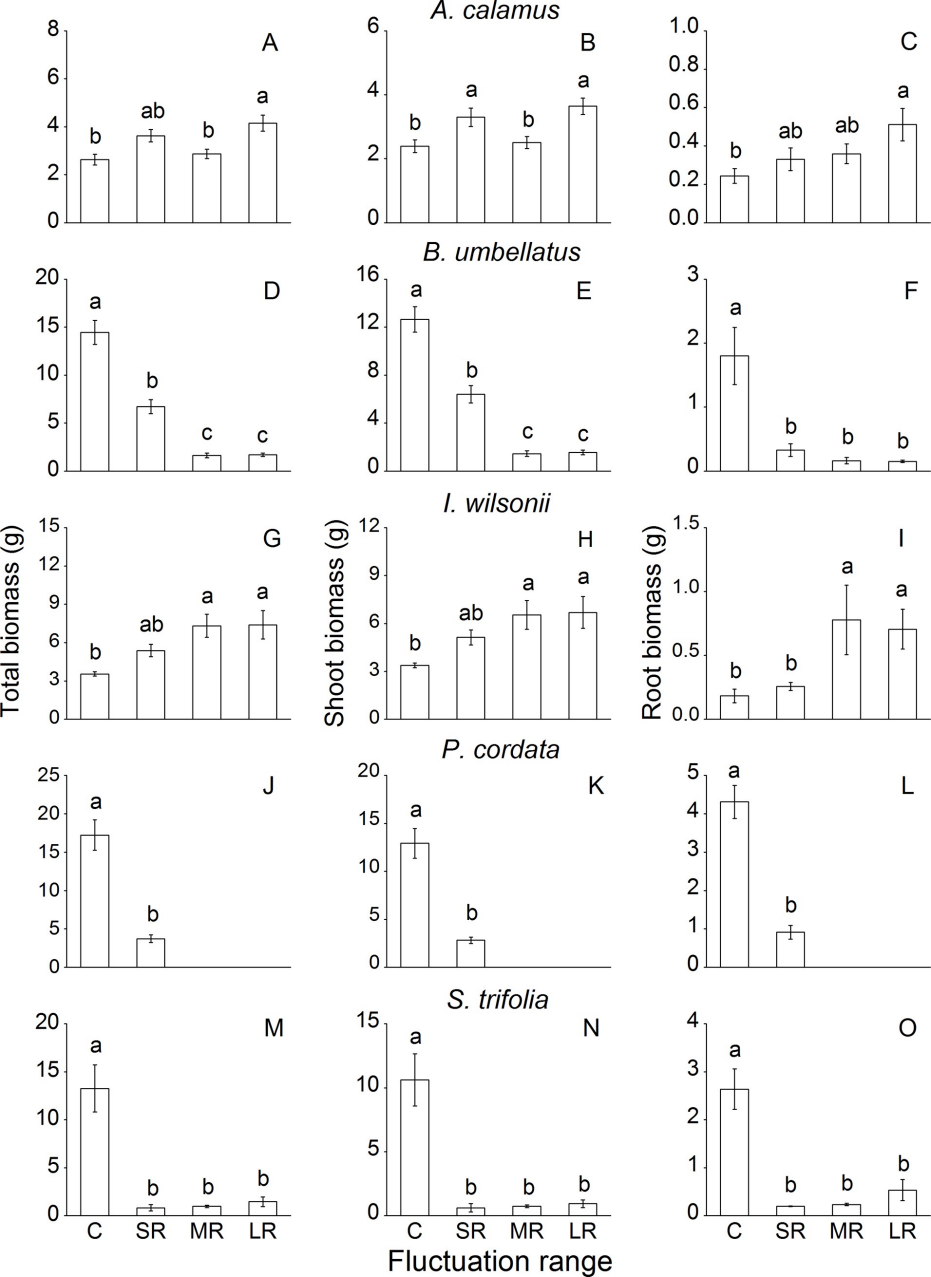
Total biomass, shoot biomass and root biomass of eight common riparian plants after 80-d treatments with different ranges of water level fluctuation. For each species, means of treatments with different letters are significantly different at *P* = 0.05. Data are mean values ± s.e.

Fluctuation range significantly affected shoot biomass, root biomass and total biomass of *A*. *calamus*, *B*. *unbellatus*, *I*. *wilsonii*, *P*. *cordata* and *S*. *trifolia*, except for the root biomass of *I*. *wilsonii* (Table 2A). Biomass of *A*. *calamus* was significantly higher in LR than in the control (Fig 2A–2C). Likewise, biomass of *I*. *wilsonii* was higher in MR and LR than in the control (Fig 2G–2I). In contrast, biomass of *B*. *unbellatus*, *P*. *cordata* and *S*. *trifolia* decreased remarkably by water fluctuation at all ranges (Fig 2). Not enough plants of *L*. *salicaria*, *P*. *hydropipier* and *T*. *minima* survived for the biomass measurements on day 80.

**Table 2:** Effects of fluctuation range (A) and frequency (B), respectively, on total biomass, shoot biomass and root biomass of the eight riparian species.

	(A) Fluctuation range				(B) Fluctuation frequency			
Species	DF	Total	Shoot	Root	DF	Total	Shoot	Root
*A*. *calamus*	3,16	7.4^**^	6.5^**^	3.4^*^	3,20	4.5^*^	2.2^ns^	9.9^**^
*B*. *umbellatus*	3,15	68.0^***^	68.4^***^	11.3^***^	3,17	1.6^ns^	0.9 ^ns^	4.3^*^
*I*. *wilsonii*	3,18	4.8^*^	3.9^*^	3.2^ns^	3,15	40.0^***^	18.9^***^	38.8^***^
*L*. *salicaria*	-	-	-	-	2,13	1.7^ns^	1.4^ns^	1.9^ns^
*P*. *cordata*	1,4	71.5^**^	68.7^**^	73.9^**^	3,18	8.1^**^	5.2^*^	8.7^**^
*P*. *hydropiper*	-	-	-	-	-	-	-	-
*S*. *trifolia*	3,11	23.2^***^	22.5^***^	24.0^***^	2,13	15.6^**^	76.1^***^	4.7^*^
*T*. *minima*	-	-	-	-	-	-	-	-

*F* values and significance levels (***P < 0.001, **P < 0.01, *P < 0.05 and ^ns^*P* ≥ 0.05) of one-way ANOVA are given. DF: degrees of freedom; -: data are not available due to low number of surviving plants.

**Fig 3:**
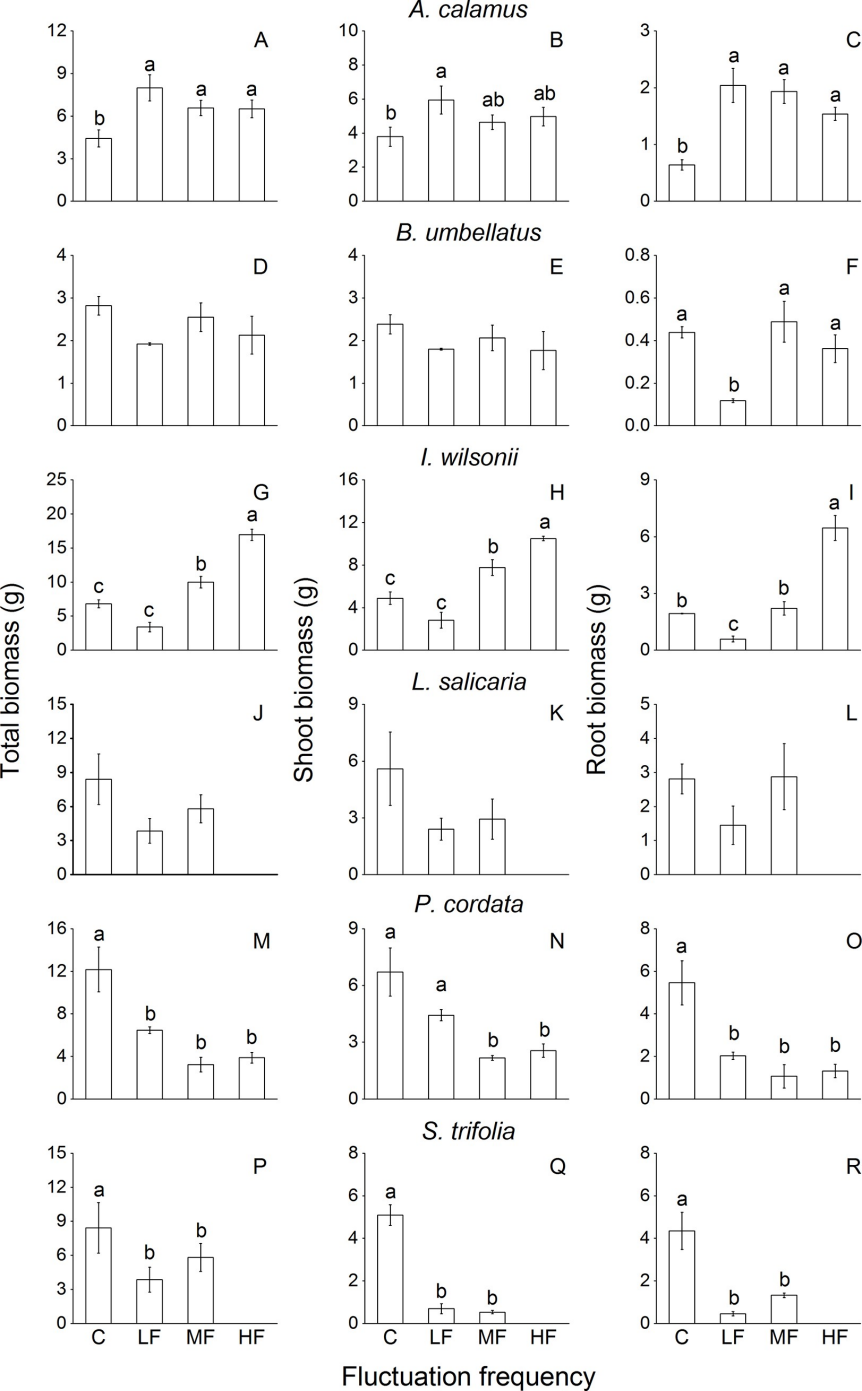
Total biomass, shoot biomass and root biomass of eight common riparian plants after 80-d treatments with different frequencies of water level fluctuation. For each species, means of treatments with different letters are significantly different at *P* = 0.05. Data are mean values ± s.e.

Fluctuation frequency significantly affected root biomass and total biomass of *A*. *calamus*, root biomass of *B*. *umbellatus*, and shoot biomass, root biomass and total biomass of *I*. *wilsonii*, *P*. *cordata* and *S*. *trifolia* (Table 2B). Total biomass and root biomass of *A*. *calamus* were significantly higher in the treatments with water fluctuation at all frequencies than in the control, and its shoot biomass was also higher in LF than in the control (Fig 3A–3C). Total biomass and shoot biomass of *I*. *wilsonii* were higher in MF and HF than in the control, and its root biomass was also higher in HF than in the control (Fig 3G–3I). Total biomass and shoot biomass of *B*. *umbellatus* and *L*. *salicaria* were not significantly affected by fluctuation frequency, except for *L*. *salicaria* in HF (Fig 3D, 3E, 3J and 3K). However, fluctuation at different frequencies markedly decreased biomass of *P*. *cordata* and *S*. *trifolia* (Fig 3M–3R). As in Exp. 1, biomass data of *P*. *hydropiper* and *T*. *minima* were not available for Exp. 2 due to the low survival rates of these plants.

### Biomass allocation

**Fig 4:**
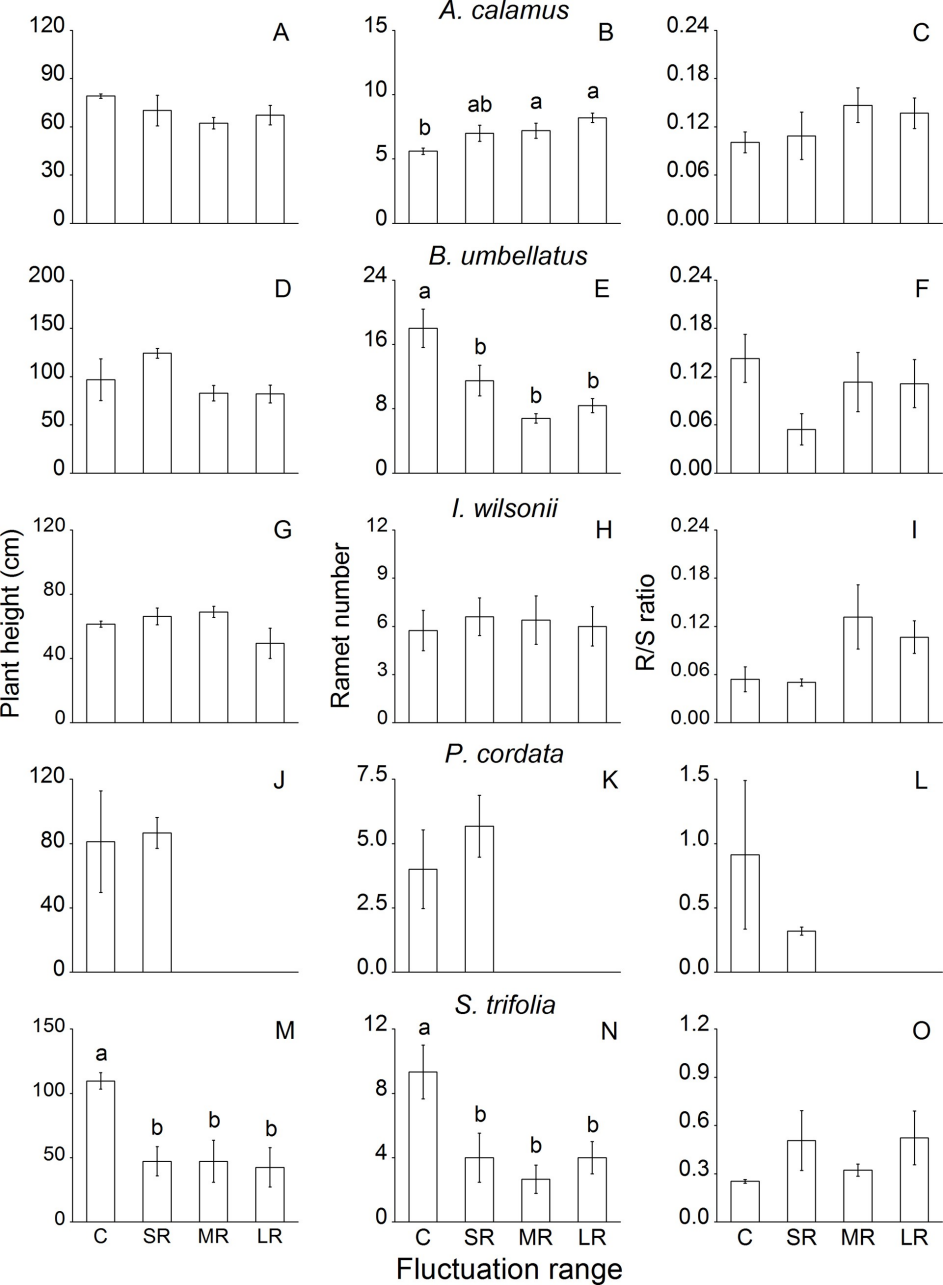
Plant height, ramet number and root-to-shoot (R/S) ratio of eight common riparian plants after 80-d treatments with different ranges of water level fluctuation. For each species, means of treatments with different letters are significantly different at *P* = 0.05. Data are mean values ± s.e.

Fluctuation range significantly affected ramet number of *A*. *calamus* and *B*. *unbellatus*, and plant height and ramet number of *S*. *trifolia* (Table 3A). Ramet number of *A*. *calamus* was significantly higher in MR and LR than in the control (Fig 4B). However, ramet numbers of *B*. *umbellatus* and *S*. *trifolia* were lower in fluctuation at all ranges than in the control (Fig 4E and 4N). Fluctuation at all ranges significantly decreased plant height of *S*. *trifolia* compared to the control (Fig 4M).

**Table 3:** Effects of fluctuation range (A) and frequency (B), respectively, on plant height (PH), ramet number (RN) and root to shoot ratio (R/S) of the eight riparian species.

	(A) Fluctuation range				(B) Fluctuation frequency			
Species	DF	PH	RN	R/S	DF	PH	RN	R/S
*A*. *calamus*	3,19	1.4^ns^	4.9^*^	1.1^ns^	3,20	0.9^ns^	2.5^ns^	5.1^*^
*B*. *umbellatus*	3,18	2.0^ns^	10.3^**^	1.3^ns^	3,17	14.0^***^	1.3^ns^	2.6^ns^
*I*. *wilsonii*	3,18	2.1^ns^	0.1^ns^	1.4^ns^	3,15	2.1^ns^	1.9^ns^	3.5^*^
*L*. *salicaria*	-	-	-	-	2,13	4.5^*^	0.1^ns^	1.7^ns^
*P*. *cordata*	4	-0.2^ns^	-0.9^ns^	1.0^ns^	3,18	10.2^**^	2.1^ns^	2.2^ns^
*P*. *hydropiper*	-	-	-	-	-	-	-	-
*S*. *trifolia*	3,11	6.1^*^	5.1^**^	1.1^ns^	2,13	22.2^***^	1.1^ns^	4.8^*^
*T*. *minima*	-	-	-	-	-	-	-	-

*F* values and significance levels (***P < 0.001, **P < 0.01, *P < 0.05 and ^ns^*P* ≥ 0.05) of one-way ANOVA are given. DF: degrees of freedom; -: data are not available due to low number of surviving plants.

**Fig 5:**
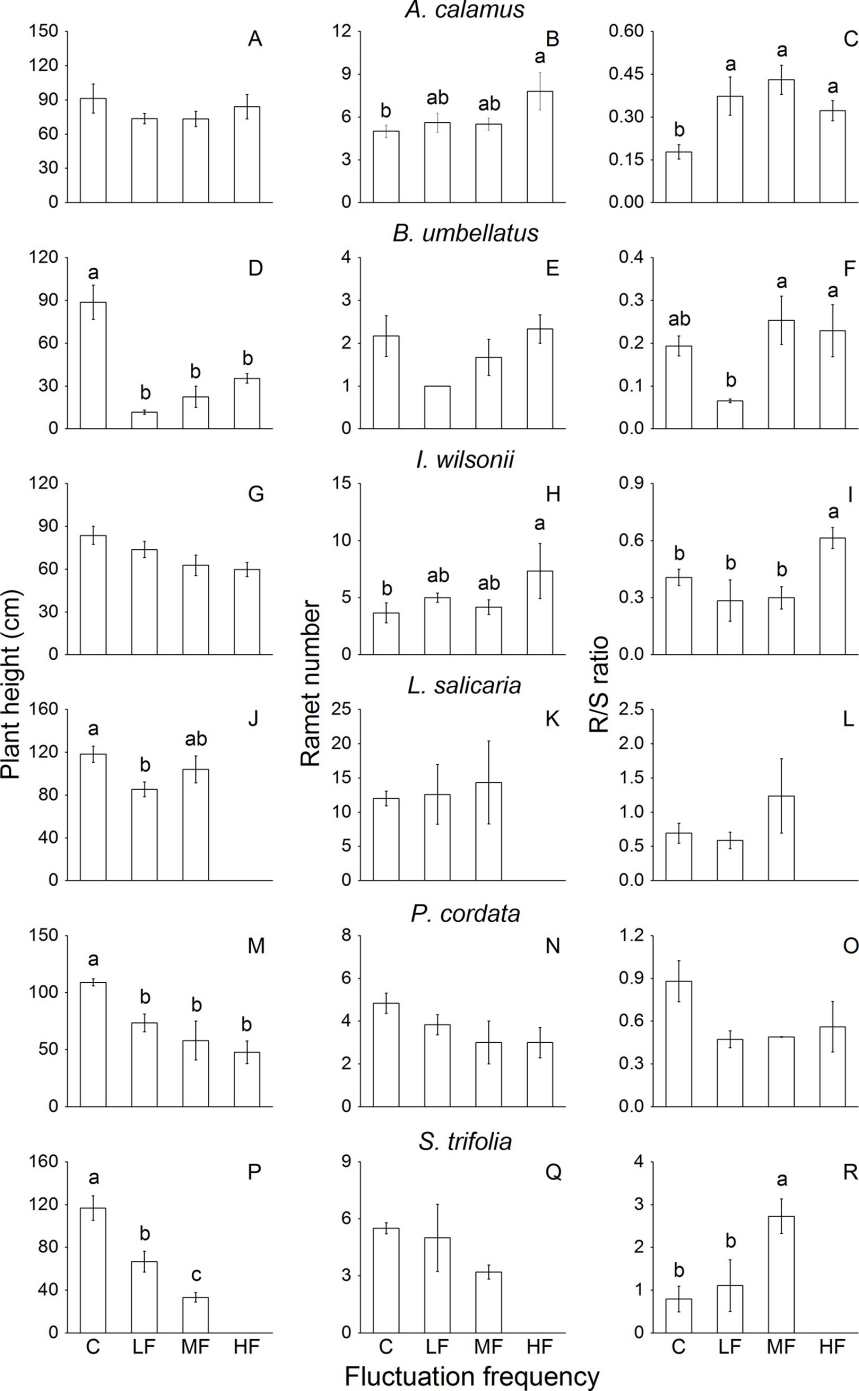
Plant height, ramet number and root-to-shoot (R/S) ratio of eight common riparian plants after 80-d treatments with different frequencies of water level fluctuation. For each species, means of treatments with different letters are significantly different at *P* = 0.05. Data are mean values ± s.e.

Fluctuation frequency significantly affected plant height of *B*. *umbellatus*, *L*. *salicaria*, *P*. *cordata* and *S*. *trifolia*, and root-to-shoot ratio (R/S) of *A*. *calamus*, *I*. *wilsonii* and *S*. *trifolia* (Table 3B). Compared to the control, fluctuation at all frequencies significantly decreased plant height of *B*. *umbellatus*, *P*. *cordata* and *S*. *trifolia* (Fig 5D, 5M and 5P). The HF treatment increased ramet number of *A*. *calamus* and *I*. *wilsonii* (Fig 5B and 5H). Fluctuation at all frequencies significantly increased R/S of *A*. *calamus* while HF and MF increased R/S of *I*. *wilsonii* and *S*. *trifolia*, respectively (Fig 5C, 5I and 5R).

## Discussion

*A*. *calamus*, *B*. *umbellatus*, *I*. *wilsonii* and *S*. *trifolia* showed high survival rates when subjected to water fluctuation with different ranges (Table 1A). As for fluctuation with different frequencies, *A*. *calamus*, *B*. *umbellatus*, *I*. *wilsonii* and *P*. *cordata* had high survival rates (Table 1B). The survival rates of the first four species were comparable after the 80-d treatments with different fluctuation ranges, while the survival rates of *A*. *calamus*, *B*. *umbellatus* and *I*. *wilsonii* were higher at MF than at LF and HF. This confirms the first hypothesis that intermediate levels of fluctuation frequency have beneficial effects on plant survival compared to constant submergence and low- or high-frequency fluctuation [14, 16, 22]. Our results are also consistent with the former findings that moderate levels of water fluctuation promote seed germination and seedling establishment of riparian plants, as well as growth of submerged macrophytes in wetlands [11, 13–14, 22, 28].

Acclimation to water level fluctuation is, however, not a common feature of wetland plants. In the present study, *L*. *salicaria*, *P*. *cordata*, *P*. *hydropiper* and *T*. *minima* showed low survival rates in the treatments with various fluctuation ranges (Table 1A). Compared to the other species, *L*. *salicaria* and *P*. *hydropiper* may have relatively smaller carbohydrate storage [34–35], which could be a reason for their poor survival rates. Besides, a low capacity to cope with low O_2_, low light intensity and high hydrostatic pressure could be another reason [21–22]. In response to fluctuation frequency, *L*. *salicaria*, *P*. *hydropiper*, *S*. *trifolia* and *T*. *minima* had low survival rates (Table 1B). The high frequency leads to faster changes in aerobic and hypoxia conditions [8, 16], which may be not long enough for these species to resume photosynthesis and growth in the aerobic condition, therefore resulting in a high mortality.

In response to fluctuation range, only *A*. *calamus* and *I*. *wilsonii* could maintain comparable or even higher growth and biomass accumulation than the control plants, while growth of the other six species decreased, especially in MR and LR (Fig 2). Compared with SR, the LR treatment involved deeper submergence, resulting in lower light intensity, slower gas diffusion and higher water pressure [29, 36]. The greatly decreased light intensity and gas diffusion would severely restrict photosynthesis and plant growth [37–38]. Water pressure could also be another important factor that has limited plant growth in LR and MR [35], although the effect of water pressure is still unclear. Additionally, significantly decreased ramet number found in the fluctuation range experiment could also have led to reduced growth of *B*. *umbellatus* and *S*. *trifolia* (Fig 4).

When challenged by different water fluctuation frequencies, *A*. *calamus*, *B*. *umbellatus* and *I*. *wilsonii* could maintain or even increase biomass accumulation compared with the control plants (Fig 3). Correspondingly, these species could maintain or significantly increase the ramet number during the treatments with different fluctuation frequencies (Fig 5). Maintaining or increasing the capacity for asexual propagation is important for survival of wetland plants subjected to mechanical stress caused by streaming water and wind [39–40]. Asexual propagation and biomass allocation to ramets could partly counterbalance the detrimental effect of mechanical stress on sexual propagation [41]. Moreover, frequent water level fluctuations significantly increased root biomass accumulation in *A*. *calamus* at all frequencies, *I*. *wilsonii* at HF and *S*. *trifolia* at MF (Figs 3 and 5). By promoting biomass accumulation in roots, plants could more safely store carbohydrate, and at the same time build more stable anchorage in conditions with frequent disturbances [39, 42]. Larger resource storage and better anchorage will be beneficial for the maintenance, recovery and growth of shoots and new ramets.

These species seem to have the ability to acclimate to conditions with recurrent submergence, even at high frequencies. Such acclimation, called ′stress memory′ in other studies, could increase plant tolerance to subsequent exposure to the same kind of stress [43–45]. Compared with a single stress event, some studies have shown that plants were able to induce faster and stronger responses to the same stress when applied repeatedly [43, 46]. The wetland species *Alternanthera philoxeroides* showed dynamic photosynthetic acclimation in response to cyclic events of submergence and de-submergence, with down-regulation during submergence and prompt up-regulation after de-submerged [31]. Moreover, an increasing number of evidence have been provided to demonstrate growth, photosynthetic and/or metabolic acclimation to recurrent abiotic stress, such as drought [47], cold [48], high temperature [46] and salinity [49].

Validating our second hypothesis, species with high survival rates and growth capacity neither elongated shoot nor invested relatively more biomass in shoot growth during water level fluctuation with all ranges and frequencies tested (Figs 4 and 5). Shoot growth plasticity such as stem/petiole elongation is commonly observed in wetland plants in response to continuous submergence, which is triggered by fast increase of endogenous ethylene concentration [34, 50–52]. However, we found that shoot elongation was greatly impeded in response to water level fluctuation at different ranges and frequencies (Figs 4 and 5). Recurrent de-submergence episodes during water fluctuation re-expose submerged plants to aerobic conditions. When de-submerged, the entrapped ethylene in plant tissues could be quickly lost due to its high diffusion coefficient in aerobic conditions [53]. The ethylene concentration might be difficult to reach the threshold to trigger stem and petiole elongation. Furthermore, strong sunlight and UV may also inhibit elongation growth of these organs upon exposure to aerobic conditions [54–55]. Another merit of not elongating (fragile) shoot may be the low risk of mechanical damage in floodwater when the shoot remains compact [39–40].

We conclude that the plant species with high survival rates (≥ 50%), such as *A*. *calamus*, *B*. *umbellatus* and *I*. *wilsonii*, perform better at the medium level than at the low and high levels of water fluctuation frequency. Accordingly, they can benefit from the intermediate frequency of water fluctuation. While the species differed in biomass allocation in roots and ramets, they similarly showed reduction of shoot elongation during water fluctuation at varying ranges and frequencies. Therefore, the capacity for asexual propagation and biomass accumulation rather than shoot elongation is important for survival of these plants during water fluctuation. These findings could provide some hints to develop optimal strategies for management and restoration of degraded or destroyed wetland ecosystems with frequent water fluctuation and disturbance.

## Supporting information

S1 Table General information of eight common riparian species. (PDF)
