# Supplementary material for: Growth responses of eight wetland species to water level fluctuation with different ranges and frequencies
Source: PLoS One. 2019 Jul 25;14(7):e0220231. doi: 10.1371/journal.pone.0220231 (PMC6657884; doi:10.1371/journal.pone.0220231)
Supplement: S1 Table — (PDF) [file pone.0220231.s001.pdf]

S1 Table. General information of eight common riparian species.

Species	Family	Life history	Initial biomass (g)
Acorus calamus L.	Araceae	Perennial	2.94 ± 0.38
Butomus umbellatus L.	Butomaceae	Perennial	6.34 ± 0.83
Iris wilsonii C. H. Wright	Iridaceae	Perennial	17.04 ± 1.05
Lythrum salicaria L.	Lythraceae	Perennial	4.94 ± 0.90
Polygonum hydropiper L.	Polygonaceae	Annual	0.39 ± 0.07
Pontederia cordata L.	Pontederiaceae	Perennial	4.68 ± 0.51
Sagittaria trifolia L.	Alismataceae	Perennial	2.43 ± 0.46
Typha minima Funck ex Hoppe	Typhaceae	Perennial	9.18 ± 2.60
